# The IL-1-Like Cytokine IL-33 Is Constitutively Expressed in the Nucleus of Endothelial Cells and Epithelial Cells *In Vivo*: A Novel ‘Alarmin’?

**DOI:** 10.1371/journal.pone.0003331

**Published:** 2008-10-06

**Authors:** Christine Moussion, Nathalie Ortega, Jean-Philippe Girard

**Affiliations:** 1 CNRS, IPBS (Institute of Pharmacology and Structural Biology), Toulouse, France; 2 University of Toulouse, UPS, IPBS, Toulouse, France; New York University School of Medicine, United States of America

## Abstract

**Background:**

Interleukin-33 (IL-33) is an IL-1-like cytokine ligand for the IL-1 receptor-related protein ST2, that activates mast cells and Th2 lymphocytes, and induces production of Th2-associated cytokines *in vivo*. We initially discovered IL-33 as a nuclear factor (NF-HEV) abundantly expressed in high endothelial venules from lymphoid organs, that associates with chromatin and exhibits transcriptional regulatory properties. This suggested that, similarly to IL-1α and chromatin-associated cytokine HMGB1, IL-33 may act as both a cytokine and a nuclear factor. Although the activity of recombinant IL-33 has been well characterized, little is known yet about the expression pattern of endogenous IL-33 *in vivo*.

**Methodology/Principal Findings:**

Here, we show that IL-33 is constitutively and abundantly expressed in normal human tissues. Using a combination of human tissue microarrays and IL-33 monoclonal and polyclonal antibodies, we found that IL-33 is a novel nuclear marker of the endothelium widely expressed along the vascular tree. We observed abundant nuclear expression of IL-33 in endothelial cells from both large and small blood vessels in most normal human tissues, as well as in human tumors. In addition to endothelium, we also found constitutive nuclear expression of IL-33 in fibroblastic reticular cells of lymphoid tissues, and epithelial cells of tissues exposed to the environment, including skin keratinocytes and epithelial cells of the stomach, tonsillar crypts and salivary glands.

**Conclusions/Significance:**

Together, our results indicate that, unlike inducible cytokines, IL-33 is constitutively expressed in normal human tissues. In addition, they reveal that endothelial cells and epithelial cells constitute major sources of IL-33 *in vivo*. Based on these findings, we speculate that IL-33 may function, similarly to the prototype ‘alarmin’ HMGB1, as an endogenous ‘danger’ signal to alert the immune system after endothelial or epithelial cell damage during trauma or infection.

## Introduction

IL-33 (initially designated NF-HEV for “Nuclear Factor from High Endothelial Venules” [1]) is the most recent addition to the IL-1 family [2], [3]. It has been shown to function as a ligand for the IL-1 receptor-related protein ST2 (IL-1R4), a receptor expressed on mast cells, T helper type 2 (Th2) lymphocytes and cardiomyocytes [2], [4]. Accordingly, recombinant IL-33 was found to drive production of pro-inflammatory and Th2-associated cytokines in mast cells and Th2 lymphocytes [2], [5]–[10], to induce chemotaxis of Th2 cells [11] and to protect the heart against cardiac stress [4] and atherosclerosis [12]. The effects of IL-33 were found to be abrogated in the absence of the IL-1R accessory protein (IL-1RAcP), a signaling receptor subunit that is also a member of the IL-1R complex, indicating that ST2/IL-1RAcP comprise the IL-33 signaling receptor complex [7], [13].

Although the functional properties of recombinant IL-33 have been well characterized, little is known yet about endogenous IL-33 *in vivo*. Using *in situ* hybridization and immunohistochemistry with three distinct antisera, we previously reported that IL-33 is abundantly expressed in endothelial cells of high endothelial venules (HEVs), specialized blood vessels which mediate lymphocyte recruitment into lymphoid organs [1], [3]. We showed that IL-33 possesses transcriptional regulatory properties and associates with chromatin in the nucleus of HEV endothelial cells *in vivo* [3], [14]. Together, these observations suggested that IL-33 is a dual function protein that may act as both a cytokine and an intracellular nuclear factor [3], [14], [15]. A similar duality of function has previously been shown for IL-1α and chromatin-associated cytokine HMGB1. IL-1α, a cell-associated cytokine, exhibits potent pro-inflammatory cytokine activities mediated by the cell surface IL-1 receptors [16] but also functions intracellularly, by translocating to the nucleus and regulating transcription [17]. HMGB1 regulates transcription, chromatin structure and function in the nucleus [18] but also functions as a potent proinflammatory cytokine when released after tissue injury and cell necrosis [19]–[21] or actively secreted by macrophages during inflammation [22]. IL-1α and HMGB1 were thus defined as endogenous ‘danger’ signals or ‘alarmins’ that may alert the immune system after cell and tissue damage during trauma or infection [23].

So far, HEV endothelial cells constitute the only human cell type that has been shown to express endogenous IL-33 at both the mRNA and protein level *in vivo*. *In situ* hybridization studies also indicated that endothelial cells constitute a major source of IL-33 in chronically inflamed tissues from patients with rheumatoid arthritis and Crohn's disease [3]. In contrast to these *in vivo* analyses, real-time quantitative PCR (qPCR) studies in cultured cells revealed expression of IL-33 mRNA in arterial smooth muscle cells, bronchial epithelial cells and activated dermal fibroblasts and keratinocytes, but expression in endothelial cells was not reported [2]. Recently, studies in rodents indicated that IL-33 is expressed in the heart, by cardiac fibroblasts after mechanical stress [4] and by endothelial cells during atherosclerosis [12].

In the present study, we used a combination of human tissue microarrays, IL-33 monoclonal and polyclonal antibodies and blocking peptides, to analyze the expression pattern of IL-33 *in vivo*. We found that IL-33 is abundantly expressed in the nucleus of endothelial cells in most normal human tissues as well as in human tumors. In addition to the endothelium, high levels of nuclear IL-33 were also found in fibroblastic reticular cells (FRCs) of lymphoid tissues and certain types of epithelium, including skin keratinocytes and epithelial cells of the stomach. Based on these observations, we speculate that, similarly to HMGB1, IL-33 may function as an ‘alarmin’ belonging to the larger family of damage-associated molecular pattern (DAMP) molecules [23].

## Methods

### Tissue microarrays

Two different sources of human tissue microarrays were used in this study. High density normal tissue microarray slides containing 45 normal human tissues in duplicate (AccuMax Array # A103VI-Normal tissues) were purchased from ISU-ABXIS (Accurate Chemical Corp, Westbury, NY, USA). Normal tissue microarray slides that contain 31 different human tissues (Normal adult tissue I, # 401 1110) and tumor tissue microarray slides that contain 10 different human tumor tissues (Multitumor-10 organs # 401 2402) were purchased from Provitro-GmbH (Berlin, Germany). All slides were prepared with formaldehyde fixed, paraffin-embedded human tissues cut at 5 µm.

### Antibodies

IL-33 mAb (1/200, clone Nessy-1) and polyclonal antibodies (1/200, Cter1 and Cter2, nos 210-447 and 210-933) were obtained from Alexis Biochemicals. Rabbit polyclonal antibodies against CD31 (1/100, Abcam), vWF (1/100, Dako) and desmin (1/75, Abcam), rabbit mAb anti-CD3 (1/100, clone SP7, Abcam), rat IgM mAb MECA-79 (1/200, Pharmingen), and mouse mAbs against alpha smooth-muscle cell actin (1/100, clone 1A4, Dako), fascin (1/100, clone 55K2, Dako) and CD68 (1/200, clone PG-M1, Dako), were used as primary antibodies in double staining experiments. Goat polyclonal antibodies anti-rabbit (1/200, Cy3, Amersham; Alexa 488, Molecular Probes) or anti-mouse (1/200, Cy3 or Cy2, Amersham; Biotin, Vector Laboratories) were used as secondary reagents.

### Immunohistochemistry and immunofluorescence staining

5 µm paraffin sections were deparaffinized in Histo-clear (National Diagnostics, MERCK Eurolab s.a) and rehydrated in graded alcohol series. For staining revealed through peroxydase activity, endogenous peroxydase was inhibited using 0.03% H_2_O_2_ in Methanol, 30 mn. The sections were then washed in distilled water and boiled in a microwave oven for epitope retrieval in Sodium Citrate buffer (10 mM pH 6, 20 mn). Slides were equilibrated in PBS and incubated with blocking solution (PBS, Goat serum 5%, BSA 5%) 1 h at room temperature. When biotinylated secondary antibody was used, endogenous biotin was inhibited using an avidin/biotin blocking kit from Vector. Primary antibodies were diluted in PBS, BSA 1% and incubated overnight at 4°C. For immunofluorescence detection, the sections were washed in PBS for 30 mn and incubated with secondary antibodies coupled to Cy3 or Cy5 diluted in PBS, BSA 1%, 1 h at room temperature. The sections were then washed in PBS for 30 mn and counterstained with Dapi and mounted in Mowiol embedding medium. For immunoperoxydase detection, the sections were washed in PBS for 30 mn and incubated with biotinylated secondary antibodies diluted in PBS, BSA 1%, 1 h at room temperature. The sections were then washed in PBS for 30 mn and incubated with Streptavidin peroxydase (Dako) diluted 1/100 in PBS, BSA 1% for 45 mn at room temperature. Peroxydase activity was revealed using DAB substrate (Sigma), slides were counterstained with Methyl Green (Sigma) or Hematoxylin (Sigma), then dehydrated and mounted in Safemount embedding medium (Labonord, France). For peptide-blocking experiments, the IL-33 mAb and polyclonal antibodies were adsorbed with control or IL-33 peptide (no 522-098, Alexis Biochemicals) for 1 h at 37°C.

### Image acquisition and processing

Fluorescent images were visualized using an inverted microscope Eclipse TE300 Nikon with 40X/0.75 and 100X/0.5–1.3 objectives at room temperature and captured through a DXM 1200 digital camera using Nikon ACT1 software. Brightfield images were visualized using an Eclipse 80i Nikon microscope with 40X/0.75 and 100X/1.30 objectives at room temperature and captured through a Digital Sight DS 5 M L1 Nikon camera using DS 5 M L1 Nikon software. All images were processed using Adobe Photoshop CS2 software.

## Results

### IL-33 is constitutively expressed in both HEVs and FRCs networks from human secondary lymphoid tissues

**Figure 1:**
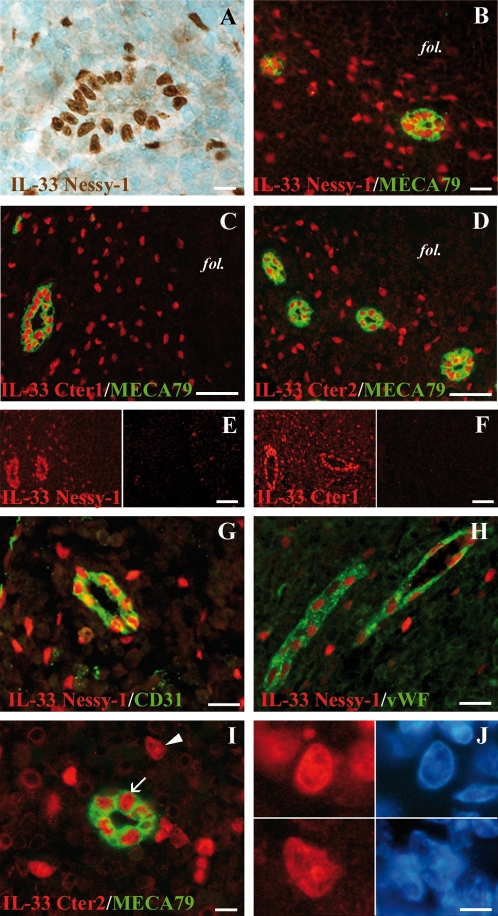
IL-33 is a chromatin-associated nuclear factor constitutively expressed in human secondary lymphoid tissues by HEVs and isolated cells in the interfollicular T cells areas. A: Immunohistochemical staining of a human tonsil section with IL-33 mAb Nessy-1. B–D: Double staining of human tonsils sections with HEV-specific mAb MECA79 (green) and IL-33 mAb Nessy-1 (B, red) or two distinct IL-33 polyclonal antisera, Cter1 (C, red) or Cter2 (D, red). The follicles (fol) are indicated. E and F: Nuclear staining of HEVs and isolated cells in the T cell areas was abrogated by pre-incubating the IL-33 monoclonal (E) and polyclonal (F) antibodies with IL-33 peptides but not control peptides. G and H: Nuclear accumulation of IL-33 in HEV blood vessels and isolated cells was also observed in lymph node (G, IL-33 mAb, red; CD31, green) and appendix (H, IL-33 mAb, red; vWF, green). I and J: Higher magnification of a human tonsil section double-stained with IL-33 and MECA-79 antibodies and counterstained with the DNA-binding dye DAPI. In the nucleus of both HEVs (arrow, upper panel) and isolated cells (arrowhead, lower panel), IL-33 accumulates in nuclear domains that colocalize with dense regions of DAPI staining (J), indicating association with chromatin. Magnification bars: A, I 10 µm; B, G, H 20 µm; J 5 µm; C, D, E, F 60 µm.

A mouse mAb against human IL-33 (mouse IgG1, clone Nessy), suitable for immunohistochemical staining, recently became available. Immunohistochemical and immunofluorescence staining of human tonsil sections with this mAb confirmed the abundant expression of IL-33 in the nucleus of HEV endothelial cells (Figure 1, A and B), as expected [3]. Interestingly, nuclear staining of scattered single cells in the interfollicular T cell areas was also observed. This staining was specific since, it was also observed with the anti-IL-33 rabbit polyclonal antibodies Cter1 and Cter2 (Figure 1, C and D) and it was abrogated by pre-incubating the IL-33 mAb and polyclonal antibodies with recombinant IL-33 peptide (Figure 1, E and F). Moreover, staining of HEV blood vessels and isolated cells in the interfollicular T-cell areas was also observed in other secondary lymphoid tissues, such as lymph nodes (Figure 1G) and appendix (Figure 1H). Interestingly, the localization of IL-33 in the nuclei of the isolated cells was not homogenous and, similarly to HEVs, higher magnification revealed co-localization with chromatin-rich domains that contained high local concentrations of DNA (Figure 1, I and J).

**Figure 2:**
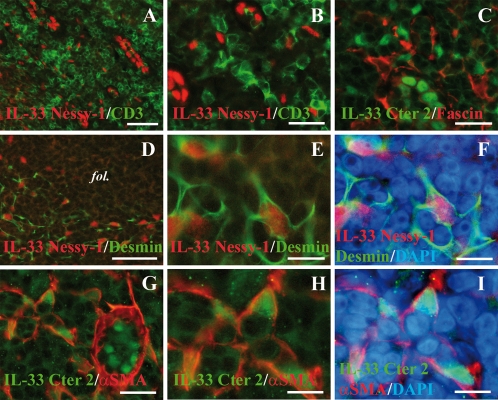
IL-33 is a nuclear marker of FRCs in the interfollicular T cells areas. A and B: Double staining of human tonsils sections with IL-33 mAb Nessy-1 (red) and anti-CD3 polyclonal antibody (green). C: Double staining of a human tonsil section with IL-33 polyclonal antibody Cter2 (green) and anti-fascin mAb (red). D–F: Double staining of a human tonsil section with IL-33 mAb Nessy-1 (red) and anti-desmin polyclonal antibody (green). A follicle is indicated (fol). Higher magnification (E) and DAPI counterstaining (F) are shown to reveal nuclear accumulation of IL-33 in desmin^+^ cells. G–I: Double staining of a human tonsil section with IL-33 polyclonal antibody Cter2 (green) and anti-α-SMA mAb (red). α-SMA is detected in the basal lamina of an HEV blood vessel (G) but is not expressed by the HEV endothelial cells. Higher magnification (H) and DAPI counterstaining (I) reveals nuclear accumulation of IL-33 in α-SMA^+^ cells. Magnification bars: A, D 60 µm; B, C, G 25 µm; E, F, H, I 10 µm.

In an effort to identify the isolated cells in the T cell areas, we then performed double staining of human tonsil sections with IL-33 mAb or polyclonal antibodies, and antibodies against T cells (CD3, Figure 2, A and B), dendritic cells (fascin, Figure 2C) or macrophages (CD68, data not shown). However, we did not observe colocalization of IL-33 with any of these markers. We next examined the possibility that these cells may correspond to stromal cells. Strikingly, double staining with an antibody against desmin, a marker of FRCs [24], [25], revealed accumulation of IL-33 in the nucleus of desmin^+^ FRCs (Figure 2, D–F). This was confirmed using an antibody against α−smooth muscle actin (α−SMA), a marker of myofibroblasts and FRCs [24] (Figure 2, G–I). It is important to note, however, that the desmin^+^ FRCs and the α−SMA^+^ FRCs did not always overlap, and IL-33 accumulated in the nucleus of many, but not all, FRCs from human tonsils. We concluded from these studies that IL-33 is a chromatin-associated nuclear factor constitutively expressed in HEVs and FRCs from human secondary lymphoid tissues.

### IL-33 is a nuclear marker of the endothelium with widespread expression along the vascular tree

**Figure 3:**
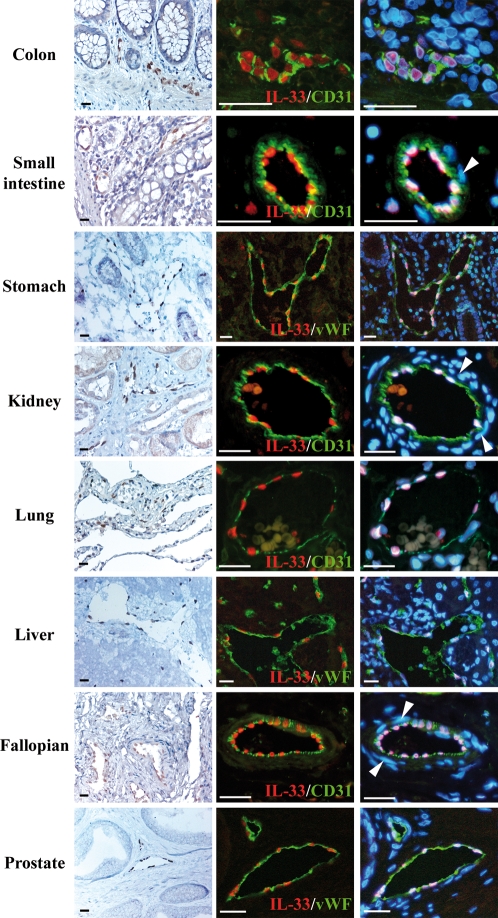
IL-33 is constitutively expressed in the nucleus of endothelial cells from large blood vessels in normal human tissues. Expression of IL-33 in human tissues was analyzed using both immunohistochemistry (lefts panels) and immunofluorescence staining (right panels). Double staining was performed with IL-33 mAb Nessy-1 (red) and anti-CD31 or anti-vWF polyclonal antibodies (green). DNA was counter-stained with DAPI. Arrowheads label the nuclei of smooth muscle cells in arterioles that are not stained with IL-33 antibodies. Magnification bars: 35 µm.

**Figure 4:**
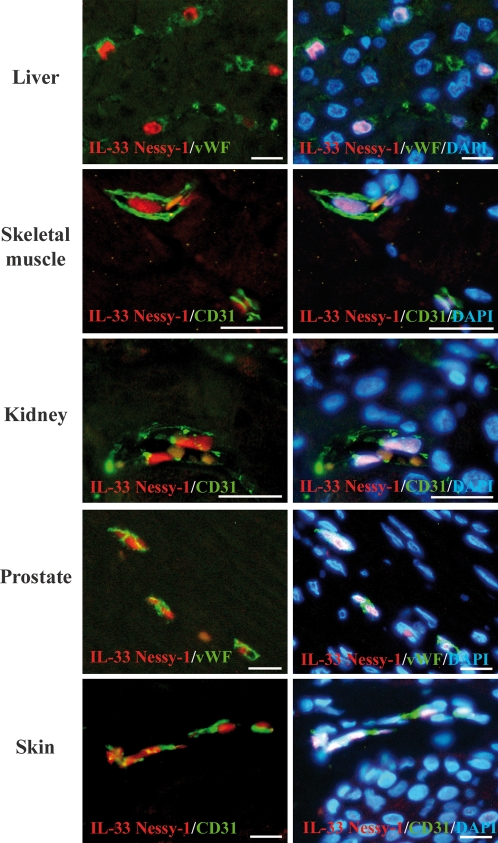
IL-33 is constitutively expressed in the nucleus of endothelial cells from small blood vessels in normal human tissues. Expression of IL-33 in the microvasculature was analyzed using immunofluorescence staining. Double staining was performed with IL-33 mAb Nessy-1 (red) and anti-CD31 or anti-vWF polyclonal antibodies (green). DNA was counter-stained with DAPI. Magnification bars: 20 µm.

To determine the expression profile and potential cellular sources of IL-33 in non-lymphoid tissues, we stained human tissues microarrays (>50 human tissues) with IL-33 antibodies. We used two independent sources of tissue arrays to obtain a *bona fide* expression profile independent of the individuals analyzed and the type and method of preparation of the microarrays. Interestingly, this analysis revealed constitutive and widespread expression of IL-33 in the endothelium from normal human tissues (Figure 3). Specific accumulation of IL-33 in the nucleus of endothelial cells from large blood vessels was found in most tissues, as revealed by double staining with antibodies against endothelial cell markers CD31 or von willebrand factor (vWF). Similarly to the staining of HEVs and FRCs in lymphoid tissues, staining of endothelial cells nuclei with IL-33 antibodies in non-lymphoid tissues was specific since it was observed with both IL-33 mAb and polyclonal antibodies, and it was abrogated by pre-incubating the antibodies with recombinant IL-33 (data not shown). In contrast to the endothelial cells that constitutively expressed high levels of IL-33 protein, vascular smooth muscle cells from arterial blood vessels did not express IL-33 *in vivo* (Figure 3, arrowheads), despite the fact IL-33 mRNA expression has previously been detected in arterial SMCs in culture [2]. In the microvasculature of many tissues, including liver, skeletal muscle, kidney (peritubular capillaries), prostate and skin, expression of IL-33 was observed in the nucleus of endothelial cells from small blood vessels (Figure 4). However, some heterogeneity was found since IL-33 was not detected in the microcirculation of the brain and kidney glomeruli (data not shown). Together, these results indicated that IL-33 is a novel nuclear marker of the endothelium with widespread expression along the vascular tree.

### IL-33 is abundantly expressed in the nucleus of endothelial cells in multiple human tumor tissues

**Figure 5:**
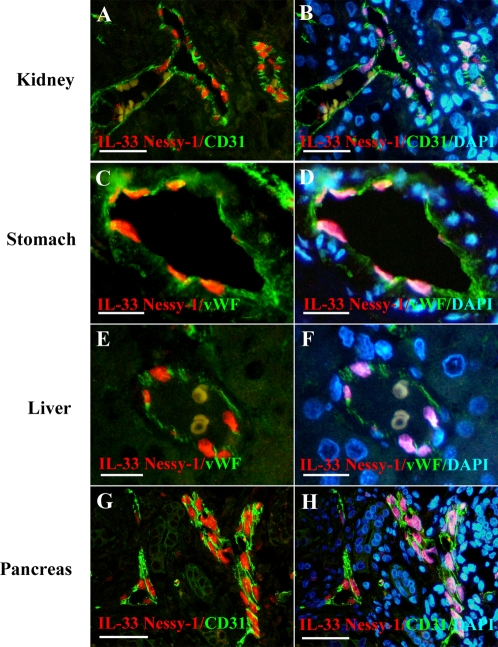
IL-33 is abundantly expressed in the nucleus of endothelial cells in human tumor tissues. Expression of IL-33 in the indicated human tumor tissues was analyzed using immunofluorescence staining. Double staining was performed with IL-33 mAb Nessy-1 (red) and anti-CD31 or anti-vWF polyclonal antibodies (green). DNA was counter-stained with DAPI. Magnification bars: A,B,G,H 50 µm; C,D,E,F 20 µm.

We then asked whether IL-33 is expressed in blood vessels from human tumor tissues. For that purpose, we double-stained human multi-tumor tissue microarrays with antibodies against IL-33 and CD31 or vWF (Figure 5). We found abundant expression of IL-33 in the nucleus of CD31^+^ or vWF^+^ endothelial cells from blood vessels in adenocarcinomas of the kidney (Figure 5A and B), stomach (Figure 5C and D), liver (Figure 5E and F), pancreas (Figure 5G and H), lung, breast or colon (data not shown). Therefore, IL-33 appeared to be a general nuclear marker of the endothelium expressed in both normal and tumor tissues.

### IL-33 is constitutively expressed in the nucleus of epithelial cells in tissues exposed to the environment

**Figure 6:**
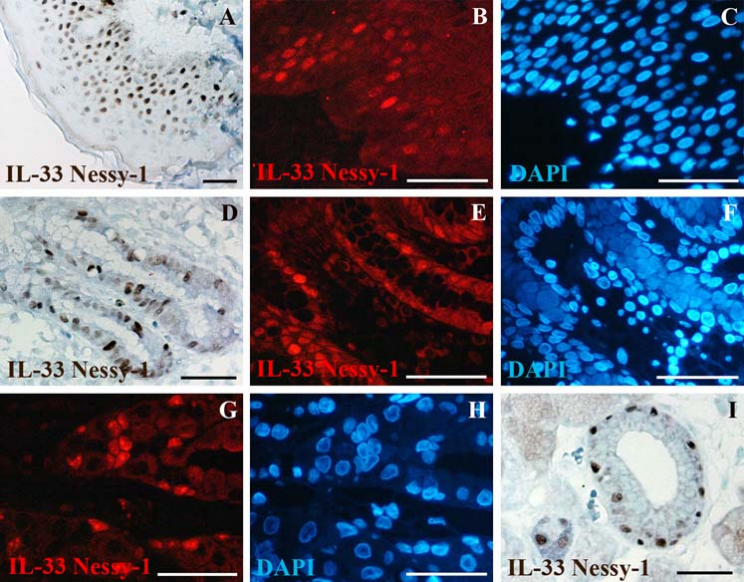
IL-33 is constitutively expressed in the nucleus of epithelial cells in tissues exposed to the environment. A–C: Skin keratinocytes; D–F: Epithelial cells of the mucosal surface in the stomach; G–H: Epithelial cells of the gastric glands in the stomach; I: Epithelial cells of the salivary glands. Immunohistochemical and immunofluorescence staining were performed with IL-33 mAb Nessy-1. For immunofluorescence, IL-33 expression was detected in red and DNA was counter-stained with DAPI (blue). Magnification bars: 70 µm.

In many tissues, IL-33 expression appeared to be restricted to endothelial cells. However, in certain tissues exposed to the environment, high levels of IL-33 were also found in the nucleus of epithelial cells. For instance, IL-33 expression was detected in skin keratinocytes (Figure 6, A–C), in epithelial cells of the mucosal surface (Figure 6, D–F) and gastric glands (Figure 6, G and H) in the stomach, as well as in epithelial cells of tonsillar crypts (data not shown) and salivary glands (Figure 6I). Although IL-33 staining in skin keratinocytes and epithelial cells of the stomach was consistently observed in all samples analyzed, significant variability was noted between different cells, between different parts of the tissues and between different individuals, suggesting modulation of IL-33 expression by local environmental cues.

As discussed above, IL-33 was not constitutively expressed in vascular smooth muscle cells *in vivo* (Figure 3). It was also not found in the nucleus of cardiac or skeletal muscle myocytes. However, nuclear expression of IL-33 was observed in visceral smooth muscle cells of the gastrointestinal and urogenital tracts, although it was less intense than in endothelial or epithelial cells (data not shown). Similarly, with the exception of FRCs in lymphoid tissues, expression of IL-33 was generally not detected in fibroblasts from normal human tissues. For instance, IL-33 was not observed in skin fibroblasts *in vivo*, despite the presence of IL-33 mRNA in activated dermal fibroblasts in culture [2].

## Discussion

Although the capacity of IL-33 to activate the ST2 receptor expressed on Th2 cells and mast cells has been well established [2], [5]–[11], very little is known yet about the cellular sources of IL-33 *in vivo*. In the present work, using human tissue microarrays and IL-33 monoclonal and polyclonal antibodies, we demonstrate that IL-33 is constitutively expressed in the nucleus of endothelial cells from both large and small blood vessels in most tissues analyzed (>50 human tissues). These observations, which extend our previous data that revealed expression of IL-33 in endothelial cells from lymphoid tissues, chronically inflamed rheumatoid arthritis synovium and Crohn's disease intestine [1], [3], demonstrate that IL-33 is a novel nuclear marker of the endothelium in both normal and chronically inflamed human tissues. Moreover, abundant expression of IL-33 was also observed in the nucleus of endothelial cells in several distinct human tumors, indicating that IL-33 is a marker of tumor blood vessels as well. Together, these findings provide further support to the possibility that IL-33 may play important roles as a chromatin-associated factor in the nucleus of endothelial cells *in vivo* [1], [3].

In addition to endothelium, we detected abundant expression of IL-33 in the nucleus of epithelial cells of tissues in contact with the environment, including the skin and gastrointestinal tract, where pathogens, allergens and other environmental agents are frequently encountered. Interestingly, constitutive expression in skin keratinocytes has previously been reported for other members of the IL-1 family, including IL-1α and IL-1β [16], [26], [27]. The constitutive expression of IL-33 in epithelial barriers of our body thus supports the possibility that, similarly to the IL-1s, it may play important roles in the response to injury or infection.

In all endothelial cells and epithelial cells that expressed IL-33 *in vivo*, the protein accumulated in the nucleus and we found no evidence for cytoplasmic, membrane or extracellular localization. Therefore, it is not yet clear how IL-33 may be released from the nucleus to exert its cytokine activities towards target cells expressing the ST2 receptor. The mature form of IL-33 has been proposed to be secreted after maturation by caspase-1 [2], but the predicted cleavage site for caspase-1 is not conserved in IL-33 orthologues and we found no evidence for IL-33 processing *in vivo* [3]. Two mechanisms have previously been shown to be involved in the release of chromatin-associated cytokine HMGB1, secretion by activated macrophages after hyperacetylation of lysine residues [22] or passive release by necrotic cells during cell damage or tissue injury [19]. Significant expression of IL-33 mRNA in human hematopoietic cells was not detected in previous qPCR studies [2], and accordingly, in the present work, we did not observe expression of IL-33 protein in CD3^+^ lymphocytes, CD68^+^ tissue macrophages or Fascin^+^ dendritic cells. Therefore, active secretion by macrophages or dendritic cells is unlikely to be a major mechanism of IL-33 release *in vivo*. An alternative and more likely possibility is that IL-33 may be released from dead or dying cells during trauma or infection, and may function, similarly to HMGB1, as an endogenous ‘danger’ signal or ‘alarmin’ [23], to alert the immune system of cell or tissue damage. In this respect, mast cells, that constitute a major cellular target of IL-33 [2], [5]–[7] and are strategically positioned closed to vessel walls and epithelial surfaces exposed to the environment (including the skin and gastrointestinal tract) [28], could play an important role in the response to the IL-33 ‘alarmin’ signal released by damaged endothelial or epithelial cells. Mast cells are widely recognized for their roles as effector cells in allergic disorders, but are also important as initiators and effectors of innate immunity [28], and IL-33 may turn out to be a critical activator of mast cells during innate immune response to pathogens. IL-33 could act alone or in concert with other mediators, such as thymic stromal lymphopoietin (TSLP), which is also expressed by inflamed skin and tonsillar crypt epithelium, and can be released by epithelial cells in response to trauma or infection [5], [29], [30].

The possibility that IL-33 may play important roles in the response to trauma and infection is further supported by the observation that increased serum levels of a soluble form of the IL-33 receptor ST2 have been associated with numerous human diseases, including sepsis and trauma [31], acute myocardial infarction [32], chronic heart failure [33], idiopathic pulmonary fibrosis [34], asthma and allergic airway inflammation [35], rheumatoid arthritis and systemic lupus erythematosus [36]. The increase in serum levels of soluble ST2 likely results from the activation (or recruitment) by IL-33 of target cells co-expressing the membrane and secreted forms of the ST2 receptor, thus providing evidence for IL-33 release (or activation) in all these diseases. Our observations that demonstrate constitutive expression of IL-33 in normal human tissues are therefore important, because they suggest IL-33 can potentially be released in any tissue after injury (after endothelial or epithelial cell damage), and this could explain why the IL-33/ST2 signaling system appears to be involved in numerous human diseases affecting many different organs.

Although IL-33 was generally not detected in fibroblasts from normal human tissues, it was found to be constitutively expressed in the nucleus of T-zone FRCs of lymphoid tissues. FRCs play critical roles in lymph node organization and function by modulating lymphocyte survival and migration within T-cell areas [25], [37]. FRCs have been found to be major sources of cytokines and chemokines, such as the pro-survival factor IL-7 and the CCR7 ligands CCL19 and CCL21 [37], [38]. Our observations indicate that T-zone FRCs also constitute a major cellular source of IL-33 in secondary lymphoid organs. An intriguing possibility is that IL-33, which exhibits chemotactic properties for Th2 lymphocytes [11], may modulate migration of Th2 lymphocytes through HEV walls and FRC networks within T-cell areas in lymph nodes during infection. T-zone FRCs have been shown to exhibit myofibroblastic features, including the expression of α-SMA that is associated with increased generation of contractile forces [25], [37], [39], [40]. The expression of IL-33 in SMA^+^ FRCs may therefore be linked to their contractile myofibroblastic phenotype. Interestingly, IL-33 expression has also been shown to be biomechanically-induced in rat cardiac fibroblasts during their conversion into myofibroblasts [4]. Specific expression in fibroblasts with myofibroblast characteristics suggests involvement of IL-33 in normal and pathological wound healing, tissue fibrosis and connective-tissue remodeling after injury, processes in which myofibroblasts play critical roles [39], [40].

Surprisingly, we did not find expression of IL-33 in vascular smooth muscle cells from arterial blood vessels in normal human tissues, although expression in these cells has previously been reported using cultured cells *in vitro* [2]. Expression in cultured cells could result from a contractile phenotype biomechanically induced by culture of the cells on plastic. In support of this possibility, the mRNA encoding the canine orthologue of IL-33 (DVS27), has been found to be strongly upregulated during cerebral vasospasm, a complex pathological process characterized by prolonged contraction of arterial smooth muscle cells [41]. The apparent association of IL-33 expression with contractile forces in both smooth muscle cells and myofibroblasts [4], [41], supports an important role of IL-33 in the response to tissue injury, in addition to its roles in the response to infection. For instance, the IL-33/ST2 signaling system may be particularly important in the heart response to stress and injury during cardiovascular diseases [4], [32], [33], [42].

In summary, the results presented in this manuscript are important because they demonstrate constitutive expression of IL-33 in the nucleus of endothelial cells and epithelial cells in normal human tissues. Based on this tissue distribution, we speculate that IL-33 may function as an endogenous ‘alarmin’, similarly to chromatin-associated cytokine HMGB1, to alert the immune system of tissue injury or infection. Future studies should aim at determining the mechanisms of IL-33 release and presentation to the IL-33 signaling receptor complex, and defining the precise roles of IL-33 in human health and disease, including its functions at the level of chromatin in the nucleus of endothelial cells *in vivo*.
